# Differential expression profiling of *ΔlitR* and *ΔrpoQ* mutants reveals insight into QS regulation of motility, adhesion and biofilm formation in *Aliivibrio salmonicida*

**DOI:** 10.1186/s12864-019-5594-4

**Published:** 2019-03-15

**Authors:** Miriam Khider, Erik Hjerde, Hilde Hansen, Nils Peder Willassen

**Affiliations:** 1 Norwegian Structural Biology Centre, UiT - The Arctic University of Norway, N-9037 Tromsø, Norway; 2 Centre for Bioinformatics, Department of Chemistry, Faculty of Science and Technology, UiT - The Arctic University of Norway, N-9037 Tromsø, Norway

**Keywords:** *Aliivibrio salmonicida*, LitR, RpoQ, High cell density, Low cell density, Differentially expressed genes and quorum sensing

## Abstract

**Background:**

The coordination of group behaviors in bacteria is achieved by a cell-cell signaling process called quorum sensing (QS). QS is an intercellular communication system, which synchronously controls expression of a vast range of genes in response to changes in cell density and is mediated by autoinducers that act as extracellular signals. *Aliivibrio salmonicida,* the causative agent of cold-water vibrosis in marine aquacultures, uses QS to regulate several activities such as motility, biofilm formation, adhesion and rugose colony morphology. However, little is known about either genes or detailed mechanisms involved in the regulation of these phenotypes.

**Results:**

Differential expression profiling allowed us to define the genes involved in controlling phenotypes related to QS in *A. salmonicida* LFI1238. RNA sequencing data revealed that the number of expressed genes in *A. salmonicida, ΔlitR* and *ΔrpoQ* mutants were significantly altered due to changes in cell density. These included genes that were distributed among the 21 functional groups, mainly presented in cell envelope, cell processes, extrachromosomal/foreign DNA and transport-binding proteins functional groups. The comparative transcriptome of *A. salmonicida* wild-type at high cell density relative to low cell density revealed 1013 genes to be either up- or downregulated. Thirty-six downregulated genes were gene clusters encoding biosynthesis of the flagellar and chemotaxis genes. Additionally we identified significant expression for genes involved in acyl homoserine lactone (AHL) synthesis, adhesion and early colonization. The transcriptome profile of *ΔrpoQ* compared to the wild-type revealed 384 differensially expressed genes (DEGs) that allowed us to assign genes involved in regulating motility, adhesion and colony rugosity. Indicating the importance of RpoQ in controlling several QS related activities. Furthermore, the comparison of the transcriptome profiles of *ΔlitR* and *ΔrpoQ* mutants, exposed numerous overlapping DEGs that were essential for motility, exopolysaccharide production via *syp* operon and genes associated with *tad* operon.

**Conclusion:**

Our findings indicate previously unexplained functional roles for LitR and RpoQ in regulation of different phenotypes related to QS. Our transcriptome data provide a better understanding of the regulation cascade of motility, wrinkling colony morphology and biofilm formation and will offer a major source for further research and analysis on this important field.

**Electronic supplementary material:**

The online version of this article (10.1186/s12864-019-5594-4) contains supplementary material, which is available to authorized users.

## Background

Quorum sensing (QS) is a cell to cell communication process that allows bacteria to adjust gene expression in response to cell density [1]. The communication in QS depends on the production, accumulation and detection of signaling autoinducers such as acyl homoserine lactone (AHL) [2]. QS regulates a number of traits such as motility, biofilm formation, colonization, adhesion, virulence factor secretion and bioluminescence, which are required for survival and/or virulence in several bacteria [1]. The QS controlled activities, become costly when undertaken by an individual bacterium and are more beneficial when carried out by a group. Therefore, the QS system allows bacteria to switch between two states of gene expression: the low cell density (LCD) favoured for individuals and high cell density (HCD) favoured for groups [3–6].

*Vibrio,* species including the fish pathogen *Aliivibrio salmonicida*, are gram-negative, rod-shaped bacteria that live in different aqueous environments, including marine and freshwater [7]. *Vibrios* are known to regulate gene expression using QS system [8]. *A. salmonicida* possesses two QS systems the LuxI/R and AinS/R which are responsible for the production of eight AHLs in a cell density dependent manner [9].

Numerous studies have shown the ability of *Vibrio* species to move using flagella, mediating their movement to favorable environments and avoiding harmful conditions [10, 11]. When facing unfavorable conditions, bacteria can escape by forming biofilms [12]. A biofilm is a structured microbial community, which serves as a reservoir protecting the bacteria from being destroyed by external treatments, as well as being the main approach for survival in various harsh environmental conditions [13–15]. The development of the biofilm is a complex mechanism involving several steps. In the initial step the planktonic bacterial cells attach to the abiotic or biotic surface using physical force or bacterial appendages (flagella or pilli). Following the adhesion micro-colonies form and grow further to a three-dimensional mature biofilm structure [5, 16]. The forms of mature biofilms can vary from flat to multi-layered high mushroom-like structures, where numerous factors have been shown to influence the architecture of biofilm, including motility and extracellular polymeric substance (EPS) matrix production [4, 17]. Differing from the free-living planktonic state, cells in the biofilm are embedded in an EPS matrix, which provides strength to the interaction of the bacteria in the biofilm. EPS is mainly composed of polysaccharides in addition to proteins, lipids and nucleic acids [14, 18]. The EPS loci have been identified in several pathogenic and symbiotic vibrios [14]. For example, *A. salmonicida* and *Aliivibrio fischeri* (*A. fischeri*) produces EPS-dependent biofilm and wrinkled colonies involving an 18-gene cluster known as *symbiosis polysaccharides* (*syp*) [19, 20]. In *Vibrio cholerae* (*V. cholerae*) the *vibrio polysaccharide* (*vps*) locus encodes proteins responsible for EPS production, which is associated with rugose colony morphology and three-dimensional biofilm structure [21, 22]. The regulation of EPS biosynthesis involves several transcription regulators such as QS which sense and respond in a cell density dependent manner [14]. HapR, the QS transcription regulator of *V. cholerae* regulates expression of VpsT and VpsR regulators of biofilm [23]. At LCD *hapR* is not expressed in turn both *vpsT* and *vpsR* are upregulated allowing expression of genes involved in biofilm formation. Whereas at HCD *hapR* is expressed which results in *vpsT* and *vpsR* repression, causing the downregulation of the biofilm [23–26]. Likewise LitR (a homolog of HapR) of *A. salmonicida* is a negative regulator of biofilm formation and rugosity through *syp* repression [19, 27]. Conversely, transcription regulators OpaR, LitR and SmcR of *Vibrio parahaemolyticus* (*V. parahaemolyticus*)*, A. fischeri* and *Vibrio vulnificus* (*V. vulnificus*) respectively, are positive regulators of biofilm formation and colony opacity at HCD [28–31].

In our previous studies we were able to show that the inactivation of the LitR master regulator of QS enhanced biofilm formation, rugose colony morphology, adhesiveness and motility [19, 27]. By microarray analysis we identified a number of LitR regulated genes, among these genes were genes of the *syp* operon (*VSAL_II0295-VSAL_II0312*) and *rpoQ* sigma factor (*VSAL_II0319*) homologs of the *A. fischeri syp* and *rpoQ* genes [19, 32]*.* The inactivation of the *rpoQ* gene in *A. salmonicida* LFI1238 resulted in phenotypic traits somewhat different from the *ΔlitR* [33] . The *ΔrpoQ* mutant showed reduced motility, slimy biofilm without mushroom structure and formed an early and strong rugose colony morphology [33]. Neverless we were not able to answer how LitR and RpoQ work together to regulate QS related traits. In the present study the transcriptome expression profiles of *ΔlitR* and *ΔrpoQ* mutants were compared to the isogenic *A. salmonicida* LFI1238 wild-type, in order to gain a better understanding on how LitR and RpoQ work together and to identify the major differences in the gene expression profiles associated with the modulation of the QS related activities*.* Triplicates from each mutant were grown at low temperature (8°C) and harvested at two cell densities (LCD, OD_600_ = 0.3 and HCD, OD_600_ = 1.2). Low temperatures play an important role both in the development of cold-water vibriosis and the production of AHLs [9, 27]. Previously, we were able to show that the phenotypes exhibited by *ΔlitR* and *ΔrpoQ* (rugosity and biofilm formation) were absent at temperatures above the threshold of disease development mainly above 14°C [19, 27, 33]. Moreover, the concentration of the eight known *A. salmonicida* AHLs were also declined at high temperatures (above 16°C) [9]. Additionally, we assume that changes in cell density may affect the gene expression involved in regulating phenotypes related to QS mechanism.

## Methods

### Bacterial strains, culture conditions and supplements

Bacterial strains used in this study are listed in Table 1. *A. salmonicida* LFI1238 strain and the constructed *A. salmonicida* mutants were grow on blood agar base no. 2 (Oxoid, Thermo Scientific) with a total concentration of 5% blood and 2.5% NaCl (BA2.5) or in Luria Bertani broth (Difco, BD Diagnostics) with a total concentration of 2.5% NaCl (LB2.5). *A. salmonicida* strains were cultivated from a single colony in 2 ml (LB2.5) at 12°C, 220 rpm for 2 days.

**Table 1:** Bacterial strains and plasmids used in this study

Bacterial strains or plasmids	Description	Source
*A. salmonicida*		
LFI1238	Wild-type, isolated from Atlantic cod	[36]
*ΔlitR*	LFI1238 containing an in-frame deletion in *litR*	[27]
*ΔrpoQ*	LFI1238 containing an in-frame deletion in *rpoQ*	[33]
*ΔrpoQ-sypQ*^*−*^	*ΔrpoQ* stain with an insertional disruption in *sypQ*, Cm^r^	This study
*ΔrpoQ-sypP*^*−*^	*ΔrpoQ* stain with an insertional disruption in *sypP*, Cm^r^	This study
*ΔrpoQ-sypC*^*−*^	*ΔrpoQ* stain with an insertional disruption in *sypC*, Cm^r^	This study
LFI1238*-sypQ*^*−*^	LFI1238 containing an insertional disruption in *sypQ*, Cm^r^	This study
LFI1238*-sypP*^*−*^	LFI1238 containing an insertional disruption in *sypP*, Cm^r^	This study
LFI1238*-sypC*^*−*^	LFI1238 containing an insertional disruption in *sypC*, Cm^r^	This study
LFI1238*-* pVSV102	*A. salmonicida* LFI1238 carrying pVSV102, Kn^r^	[33]
*ΔrpoQ-*pVSV102	*ΔrpoQ* carrying pVSV102, Kn^r^	[33]
*ΔrpoQ-sypQ*^*−*^*-*pVSV102	*ΔrpoQ-sypQ*^*−*^ carrying pVSV102, Kn^r^	This study
*ΔrpoQ-sypP*^*−*^*-*pVSV102	*ΔrpoQ-sypP*^*−*^ carrying pVSV102, Kn^r^	This study
*ΔrpoQ-sypC*^*−*^-pVSV102	*ΔrpoQ-sypC*^*−*^ carrying pVSV102, Kn^r^	This study
LFI1238*-sypQ*^*−*^*-*pVSV102	LFI1238*-sypQ*^*−*^ carrying pVSV102, Kn^r^	This study
LFI1238*-sypP*^*−*^*-*pVSV102	LFI1238*-sypP*^*−*^ carrying pVSV102, Kn^r^	This study
LFI1238*-sypC*^*−*^*-*pVSV102	LFI1238*-sypC*^*−*^ carrying pVSV102, Kn^r^	This study
*E. coli*		
C118λpir	Helper strain containing pEVS104	[37]
DH5αλpir	*E. coli* strain containing GFP plasmid pVSV102	[37]
Plasmids		
pNQ705-*sypQ*^−^	pNQ705 containing an internal fragment of *sypQ*^*−*^	[19]
pNQ705-*sypP*^*−*^	pNQ705 containing an internal fragment of *sypP*^*−*^	[19]
pNQ*705-sypC*^*−*^	pNQ705 containing an internal fragment of *sypC*^*−*^	[19]
pVSV102	pES213, constitutive GFP, Kn^r^	[37]
pEVS104	R6Korigin, RP4, *oriT, trb tra* and Kn^r^	[64]

The GFP constitutive plasmid pVSV102, helper plasmid pEVS104 and suicide plasmid pNQ705 were propagated in *Escherichia coli* (*E. coli*), DH5αλpir, CC118λpir and S17.1λpir respectively. The *E. coli* strains were cultivated in LB or Luria Agar (LA) containing 1% NaCl (LB1 and LA1 respectively) and incubated at 37°C and 220 rpm. The potential transconjugants were selected on BA2.5 supplemented with 2 μl/ml chloramphenicol or 150 μl/ml kanamycin.

A seawater-based medium (SWT) was used for the transcriptomics, biofilm and morphology assays. The medium consists of 5 g/L of bacto peptone (BD Biosciences), 3 g/L of yeast extract (Sigma-Aldrich) and 28 g/L of a synthetic sea salt (Instant Ocean, Aquarium Systems).

### Transcriptomics

#### Sample collection

Three biological replicates were used for all *A. salmonicida* strains. Cultures were grown from an individual colony in 2 ml LB2.5 medium at 12°C and 220 rpm for 2 days. The 2 days culture was diluted 1:20 and grown overnight before being diluted to OD_600_ = 0.05 (optical density measured at 600 nm) in a total volume of 70 ml SWT media supplemented with 2.5% sea salt. The cultures were grown further at 8°C and 220 rpm in 250 ml baffled flask. Samples (10 ml) at low cell density OD_600_ = 0.30 and (2.5 ml) at high cell density OD_600_ = 1.20 were harvested (13,000 x g, 2 min, 4°C) (Heraeus 3XR, Thermo Scientific). Samples were persevered in 5th of their volume in RNA*later* and stored at −80°C until RNA extraction.

#### Total RNA isolation and rRNA depletion

The total RNA was extracted from the cell pellets following the standard protocols by manufactures (Masterpure DNA & RNA purification kit, Epicenter). The quality of total RNA was determined using a Bioanalyzer and Total RNA nano chip (Agilent Technologies). The ribosomal rRNA was removed from the samples using Ribo-Zero rRNA Removal kit for bacteria (Illumina) following manufactures instructions. The quality of RNA after depletion was determined using Bioanalyzer and Total RNA pico chip (Agilent Technologies).

#### RNA sequencing and data analysis

The rRNA depleted samples were used to generate RNA-sequencing libraries using TruSeq strandard mRNA library prep kit (Illumina), and sequenced at the Norwegian Sequencing Center using the Illumina NextSeq 500 with mid output reagents with 75 bp read length and paired end reads.

The sequencing quality of FASTQ files was assessed using FastQC. Further analysis of the RNA-Seq data was performed using a Galaxy pipeline consisting of EDGE-pro v1.0.1 [34] and DESeq2 [35]. EDGE-pro was used to align the reads to the *A. salmonicida* LFI1238 genome [36], and to estimate gene expression. Differences in gene expression between the reference genome of *A. salmonicida* wild-type and *ΔlitR* and *ΔrpoQ* mutants were determined using DESeq2. Log2 fold changes of the genes were recalculated to × differential expression values (i.e., Δ*litR*/wt) and genes were defined as significantly differentially expressed genes (DEGs) based on a *p*-value ≤0.05 and differentially expression values (fold change values) of ≥2 × and ≤ −2 × equal to log_2_ fold ≥1 and ≤ −1. tRNA and rRNA reads was filtered out before analysis.

The sequences from this study have been deposited in the European Nucleotide Archive (www.ebi.ac.uk/ena) under study accession number PRJEB28385.

### Construction of *A. salmonicida* LFI1238 and *ΔrpoQ* double mutants

*A. salmonicida* harboring in-frame deletion in the *rpoQ* genes (*ΔrpoQ*) is described in our recent study [33]. The pNQ705-*sypQ*^*−*^, pNQ705-*sypP*^*−*^ and pNQ705-*sypC*^*−*^ plasmids used to construct the mutants were described previously [19]. The LFI1238 and *ΔrpoQ* double mutants (Table 1) were constructed by transferring the pNQ705 plasmids carrying the targeted genes (*sypQ, sypP* and *sypC*) to LFI1238 wild-type or the *ΔrpoQ* mutant by bacterial conjugation. The conjugation of *E. coli* S17λpir harboring different pNQ705 suicide constructs to recipient cells was done as described by others [19]*.* The resulting mutant strains were named LFI1238*-sypQ*^*−*^, LFI1238***-****sypP*^*−*^*,* LFI1238***-****sypC*^*−*^*, ΔrpoQ-sypQ*^*−*^*, ΔrpoQ-sypP*^*−*^ and *ΔrpoQ-sypC*^*−*^*.*

### Construction of GFP tagged *A. salmonicida* strains

The transfer of green fluorescence protein (GFP) into *A. salmonicida* was performed by tri-parental mating as described by others [37]. Briefly, the pVSV102 plasmid carrying the gene encoding for GFP and kanamycin was transferred from *E. coli* DH5α to the mutant strains (LFI1238-*sypQ*^*−*^, LFI1238-*sypP*^*−*^, LFI1238-*sypC*^*−*^, *ΔrpoQ****-****sypQ*^*−*^, *ΔrpoQ****-****sypP*^*−*^ and *ΔrpoQ****-****sypC*^*−*^) using the conjugative helper strain CC118λpir harboring pEVS104. Donor and helper cells were grown to mid-log phase (OD_600_ = 0.7) in LB1. Recipient strains (*A. salmonicida*) were grown to early stationary phase (OD_600_ = 1.2) in LB2.5. The donor, helper and recipient were harvested (13,000 x g, 1 min) and washed twice with LB1 before they were mixed in 1 to 1 ratio and spotted onto BA2.5 plates, followed by overnight incubation at 16°C. The spotted cells were resuspended in LB2.5 and incubated for 24 h at 12°C with agitation (220 rpm). The potential tagged strains were selected on BA2.5 after 5 days. The tagged strains were confirmed microscopically with Nikon Eclipse TS100.

### Static biofilm assay

The biofilm assay was performed as described previously [19]. The overnight secondary cultures were grown to an OD_600_ of 1.3 in LB2.5. The secondary cultures were further diluted 1:10 in SWT and a total volume of 300 μl was added to each well in flat-bottom, non-tissue culture-treated Falcon 24-well plates (BD, Bioscience). The plates were incubated statically at 8°C, for 72 h and the biofilm was visualized using Nikon Eclipse TS100 microscope at 10x magnification and photographed with Nikon DS-5Mc.

### Colony morphology assay

The colony morphology assay was performed as described previously [19, 33]. The overnight secondary cultures were grown to an OD_600_ of 1.2 in LB2.5. From each secondary overnight culture, a 250 μl was harvested by centrifugation, and the pellet was re-suspended in 250 μl SWT. Then, 2 μl of each culture was spotted onto SWT agar plates, and incubated at 8°C for 12 days. The colonies were viewed microscopically with Zeiss Primo Vert and photographed with AxioCam ERc5s at 4x magnification.

## Results

### Expression profiling of the *A. salmonicida* transcriptome

The total assembled transcriptome of *A. salmonicida* wild-type LFI1238 generated an average of 9.87 million reads at LCD (OD_600_ = 0.3) and 9.56 million at HCD (OD_600_ = 1.2). The average of mapped reads to the reference genome (*A. salmonicida* LFI1238) was 88.7% at LCD and 91.4% at HCD, with an average mapping coverage of 140.6 and 141.0 respectively, indicating that the transcriptome data were sufficient for further analysis (Additional file 1: Table S1). The detailed transcriptome data of *ΔlitR* and *ΔrpoQ* are listed in Table S1 in the supplementary material (Additional file 1: Table S1).

### Cell density alters the expression pattern in *A. salmonicida* wild-type

We identified one thousand and thirteen genes to be differentially expressed in a cell density dependent manner. The majority of DEGs (70%) came from chromosome I, where the essential genes are located. The comparison (wt1.2/wt0.3) list of all DEGs are given in Table S2 in the supplementary material (Additional file 2: Table S2).

**Fig. 1:**
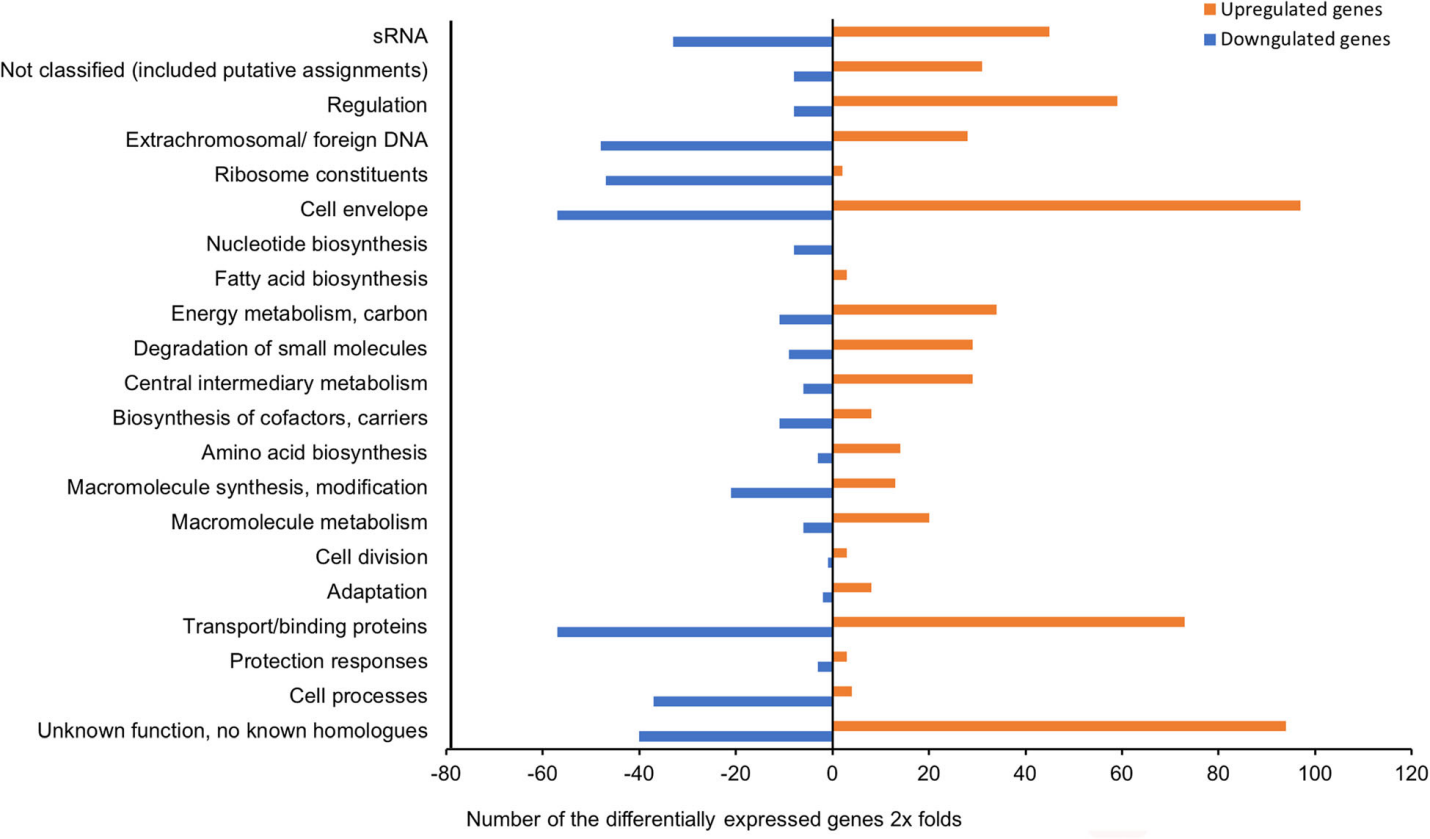
Functional distribution of genes between *A. salmonicida* wild-type at HCD compared to LCD that are ≥2 × differentially expressed

The comparison revealed that 597 (58.8%) and 416 (41.0%) of 1013 genes were significantly up- and downregulated, respectively. The 1013 DEGs were classified into different functional groups according to MultiFun [38]. Figure 1 shows a graphical presentation of the functional classes and the number of the differentially expressed genes of wild-type at HCD relative to LCD (wt1.2/wt0.3). A large number of significantly upregulated genes fell into *cell envelop* (*n* = 97, 16.2%) where the genes with highest fold change values were *VSAL_II0321* (28.25 fold-change) and *VSAL_II0322* (28.74 fold-change) encoding for putative glycosyl transferase and membrane protein, respectively. Genes with *unknown function* were next largest functional group (*n* = 94, 15.7%). Within this group, the highest fold change was observed in a number of genes coding for transposases. Among these were *VSAL_II0030* (1975.26-fold change), *VSAL_I0514* (529.36-fold-change), *VSAL_I1911* (237.06-fold change) and *VSAL_1339* (129.70-fold change). Additionally a high fold change was also observed among genes coding for arginine/ornithine periplasmic binding protein (*VASL_I1958*, 32.83-fold change) and L-amino acid binding periplasmic protein (*VSAL_I2057*, 51.77-fold change) that fell into *transport/binding proteins* functional group (*n* = 73, 12.2%).

The comparison of wild-type transcriptome at HCD relative to LCD revealed an upregulation among genes known to be associated with AHL production. The *luxI* autoinducer synthase (*VSAL_II0957*) responsible for the production of seven AHLs and its receptor *luxR1* (*VSAL_II0965*) [9], were significantly differensially expressed with a fold change values of 3.72 and 3.23, respectively.

Fifty-nine (9.8%) genes were classified into *regulation* functions, where we were able to identify the *rpoQ* sigma factor (*VSAL_II0319*) and 4 other genes from the same locus coding for putative response regulators (*VSAL_II0315*, *VSAL_II0316, VSAL_II0320, VSAL_II0329*). An additional 14 genes located close to *rpoQ* or within the same operon were also highly upregulated in the wt1.2 compared to wt0.3 and fell into other functional groups such as *cell envelope*, *extrachromosomal DNA* and *central intermediary metabolism*, in addition to some hypothetical proteins with unknown or unclassified functions. *litR*, a transcription regulator of QS (*VSAL_I2619*), was also within the 59 upregulated genes involved in regulation with a fold change of 3.43.

Even though the *fatty acid and amino acid group* showed only 3 upregulated genes, these genes exhibited high fold change values. *VSAL_I2833* coding for acetyl-coenzyme A synthetase was among the highest with 290.53-fold change value. Other highly expressed genes were grouped in *central metabolism* such as *VSAL_I2438* (57.86-fold change) and *VSAL_I2439* (61.58-fold change) coding for isocitrate lyase and malate synthase A, respectively. Among the genes that fell into a group with *not classified functions* were genes coding for putative PrkA serine protein kinase (*VSAL_I2208*, 56.64-fold change), putative anti-sigma F factor antagonist (*VSAL_II0328*, 47.87-fold change), and putative nucleotidyltransferases (*VSAL_ 2831*, 38.11-fold change) (Fig. 1 and Additional file 2: Table S2). The remaining upregulated DEGs were grouped in other functional groups (Fig. 1 and Additional file 3: Table S3).

The majority of the downregulated genes fell into *cell envelope* and *transport/ binding proteins* with 57 significantly DEGs. Among the top 5 downregulated genes within *transport/binding protein* functional group were genes of the PTS system (*VSAL_II0577*, *VSAL_II0894*, *VSAL_II0995* and *VSAL_II0966*) with fold changes ranging from −44.43 to −8.34 (Additional file 3: Table S3).

Six genes (*VSAL_II0366*, *VSLA_II0367*, *VSAL_II0368*, *VSAL_II0369*, *VSAL_II0370* and *VSAL_II0373*) located within the tight adherence (Tad) loci also known as *tad* operon were grouped in *cell envelop* and *extrachromosomal DNA* (subgroup *pathogenicity island-related functions*) functional groups. For all 6 genes the expression level ranging from −8.44 to −2.03 fold change at HCD (wt1.2) in comparison with that at LCD (wt0.3).

**Fig. 2:**
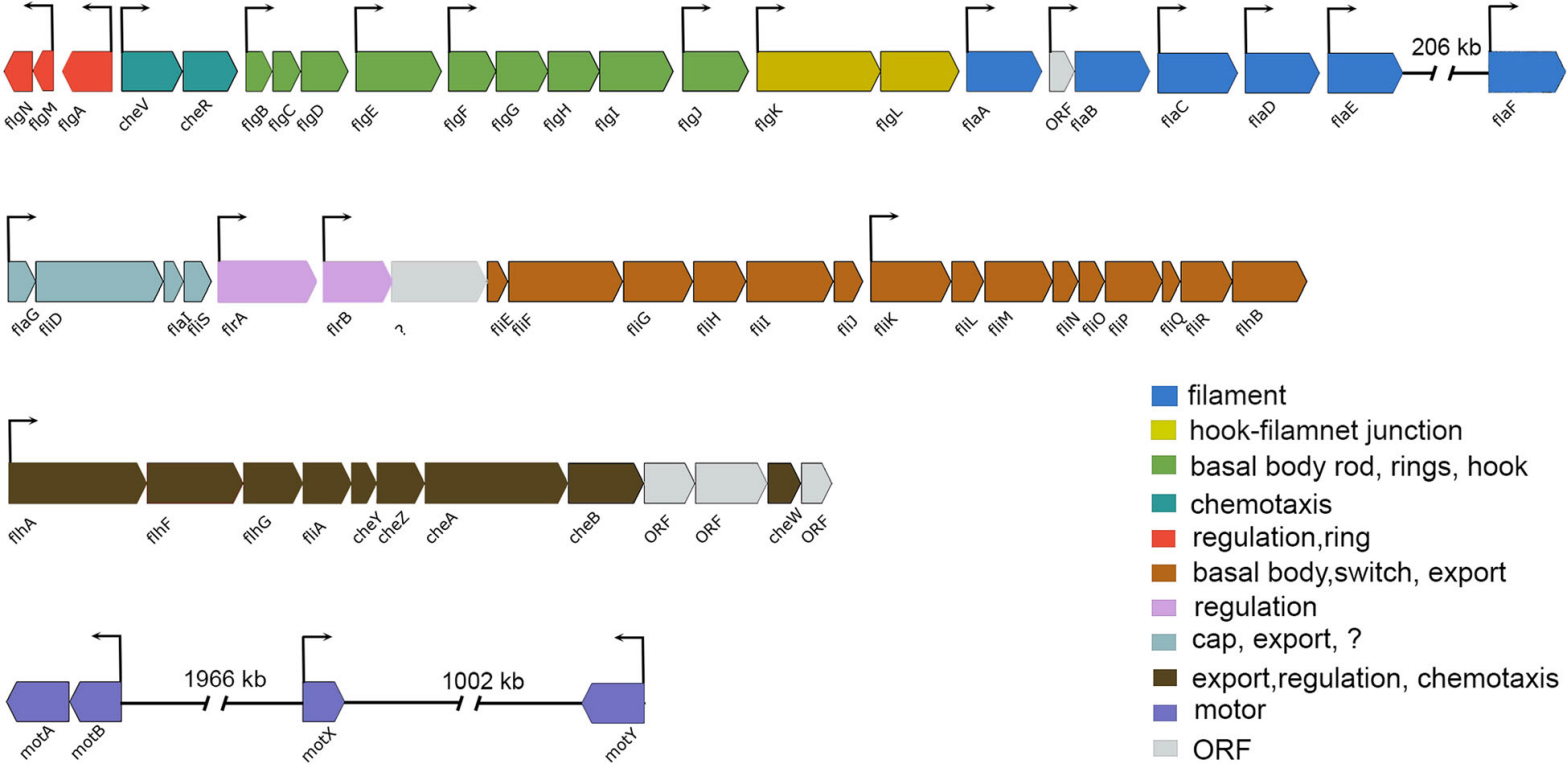
Organization of flagellar genes in *A. salmonicida* LFI1238. Arrows indicate genes and their direction of transcription. Color code provided in the image represents the different functions of each group in the flagellar apparatus. All *A. salmonicida* flagellar related genes are located on the large chromosome (first chromosome) and are organized in five chromosomal regions with clusters of flagellar genes. The six flagellin genes are located at two separated chromosomal loci. *flaABCDE* genes are found in one locus and *flaF* in a different locus. Genes encoding the flagellar motor components (*motABXY*) are located at three additional loci. Additional chemotaxis genes are scattered throughout the genome. 206 kb, 1966 kb and 1002 kb are distances between genes

Thirty-six genes out of 37 genes that fell into *cell processes* were genes involved in cell motility and chemotaxis. Figure 2 shows the organization of the flagellar genes in the *A. salmonicida* genome, and Table 2 summarizes in detail the differentially expressed genes and operons. We were able to identify 28 genes coding for flagellar components (flagellin, flagellar basal body rod, rings, hook, cap proteins and flagellar assembly proteins), 7 genes coding for methyl-accepting chemotaxis protein and one gene coding for motor component, *motY.*

**Table 2:** Thirty-six differentially expressed genes involved in motility and chemotaxis in wt1.2/wt0.3

VSAL_ID	FC	p-adjusted	Gene	Function
*VSAL_I0799*	−2.55	3.1217E-13		methyl-accepting chemotaxis protein
*VSAL_I1822*	−2.42	9.1448E-06		methyl-accepting chemotaxis protein
*VSAL_I1863*	−2.15	1.9114E-07	*motY*	sodium-type flagellar protein MotY precursor
*VSAL_I2117*	−2.26	4.7375E-06		methyl-accepting chemotaxis protein
*VSAL_I2193*	−2.96	1.4728E-16		methyl-accepting chemotaxis protein
*VSAL_I2293*	−2.18	9.8803E-11	*flhA*	polar flagellar assembly protein FlhA
*VSAL_I2295*	−2.23	1.0662E-10	*flhB*	polar flagellar assembly protein FlhB
*VSAL_I2298*	−2.17	2.498E-07	*flip*	polar flagellar assembly protein FliP
*VSAL_I2299*	−2.18	9.0124E-10	*fliO*	polar flagellar assembly protein FliO
*VSAL_I2300*	−2.11	3.8699E-09	*fliN*	polar flagellar switch protein FliN
*VSAL_I2301*	−2.02	2.1524E-08	*fliM*	polar flagellar motor switch protein FliM
*VSAL_I2302*	−2.27	1.8061E-10	*fliL*	polar flagellar protein FliL
*VSAL_I2303*	−2.05	9.5905E-09	*fliK*	polar flagellar hook-length control protein FliK
*VSAL_I2304*	−2.15	1.6591E-06	*fliJ*	polar flagellar assembly protein FliJ
*VSAL_I2305*	−2.12	2.5798E-07	*fliI*	polar flagellum-specific ATP synthase FliI
*VSAL_I2306*	−2.66	2.9148E-17	*fliH*	polar flagellar assembly protein FliH
*VSAL_I2307*	−2.61	5.6929E-15	*fliG*	polar flagellar motor switch protein FliG
*VSAL_I2308*	−2.73	7.0985E-17	*fliF*	polar flagellar M-ring protein FliF (pseudogene)
*VSAL_I2309*	−2.63	7.3748E-15	*fliE*	flagellar hook-basal body complex protein FliE
*VSAL_I2313*	−2.13	4.8203E-13	*fliS*	polar flagellar protein FliS
*VSAL_I2314*	−2.09	3.7432E-07	*flaI*	polar flagellar protein FlaI
*VSAL_I2316*	−2.06	3.2548E-06	*flaG*	polar flagellar protein FlaG (pseudogene)
*VSAL_I2319*	−2.68	1.6171E-16	*flaC*	flagellin subunit C
*VSAL_I2327*	−2.20	1.314E-05	*flaA*	flagellin subunit A
*VSAL_I2328*	−2.21	1.353E-08	*flgL*	flagellar hook-associated protein type 3 FlgL
*VSAL_I2329*	−2.33	3.2437E-10	*flgK*	hypothetical protein
*VSAL_I2330*	−2.14	5.0777E-09	*flgJ*	peptidoglycan hydrolase FlgJ
*VSAL_I2335*	−2.02	1.3131E-05	*flgE*	flagellar hook protein FlgE
*VSAL_I2336*	−2.13	1.3871E-10	*flgD*	flagellar basal-body rod protein FlgD
*VSAL_I2337*	−2.20	1.5834E-09	*flgC*	flagellar basal-body rod protein FlgC
*VSAL_I2338*	−2.29	8.8397E-09	*flgB*	flagellar basal-body rod protein FlgB
*VSAL_I2517*	−2.51	3.4203E-14	*flaF*	flagellin subunit F
*VSAL_I2897*	−2.40	6.0326E-09	*fliL*	putative flagellar basal body-associated protein FliL
*VSAL_II0675*	−2.38	0.00023876		methyl-accepting chemotaxis protein
*VSAL_II0712*	−3.87	8.4405E-30		methyl-accepting chemotaxis citrate transducer
*VSAL_II1022*	−2.60	8.1567E-05		methyl-accepting chemotaxis protein

The global comparison analysis of *A. salmonicida* wild-type at HCD compared to LCD resulted in an equal distribution of genes to be upregulated and downregulated. Additionally, the differentially expressed genes were distributed in all 21 functional classes (Fig. 1).

### Expression profiles of *A. salmonicida ΔrpoQ* and *ΔlitR* mutants compared to the wild-type at low and high cell densities

#### Expression profiling of *A. salmonicidaΔlitR* mutant

**Fig. 3:**
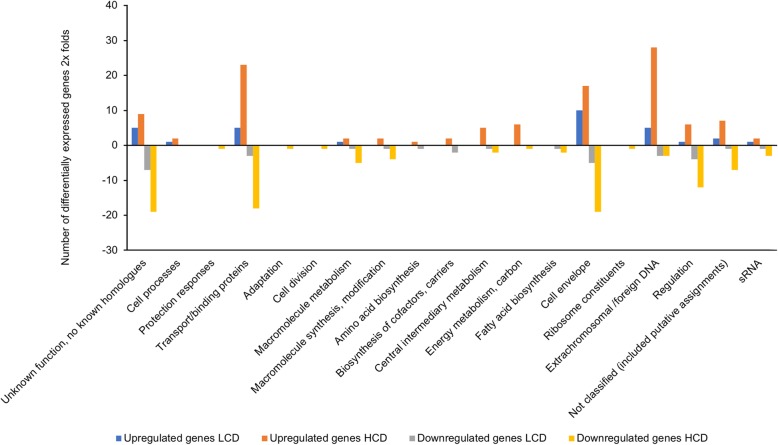
Functional distribution of genes between *A. salmonicida* wild-type and *ΔlitR* mutant at LCD and HCD that are ≥2 × differentially expressed

As shown in Fig. 3, the transcriptome of *ΔlitR* compared to the wild-type (*ΔlitR* /wt) resulted in a total of 62 DEGs at LCD, where half (*n* = 31, 50.0%) was upregulated and the other half (*n* = 31, 50.0%) was downregulated (Additional file 4: Table S4). At HCD we identified a total of 212 DEGs, 112 (53.9%) upregulated and 100 (46.0%) downregulated (Additional file 5: Table S5). The highest number of upregulated genes at LCD was represented in *cell envelope* with 10 genes (32.2%), where 4 of them were genes associated with *tad* operon. Five genes (16.1%) fell into each of *extrachromosomal/foreign DNA*, *transport/binding proteins* and genes of *unknown functions*. Other upregulated genes were involved in *transport/binding proteins*, *cell processes* mainly motility and chemotaxis, *macromolecule metabolism*, *regulation* and small RNA (*sRNA*) (Additional file 6: Table S6). The highest number of downregulated genes fell into three major groups, *unknown function* (*n* = 7, 22.5%), *cell envelope* (*n* = 5, 16.1%), and *transport/binding proteins* (*n* = 3, 9.6%). Four genes fell into the *regulation* functional group (*n* = 4, 12.9%), where the *rpoQ* sigma factor (*VSAL_II0319*) was among the significantly downregulated genes with −4.2 fold change value. The remaining downregulated genes were distributed in the other functional groups (Additional file 6: Table S6 and Fig. 3).

The 212 DEGs at HCD were distributed in 19 out of 21 functional classes (Fig. 3). The upregulated genes fell into 14 functional groups with highest number of genes in *extrachromosomal/foreign DNA*, *transport/binding proteins* and *cell envelope* groups with 28 (25%), 23 (20.5%) and 17 (15.1%) genes respectively. The downregulated genes were distributed in fewer functional groups with highest number of genes in *cell envelope* (*n* = 19), *unknown functions* (*n* = 19), *transport/ binding proteins* (*n* = 18), and *regulation* (*n* = 12). Other downregulated genes fell into other functional categories and range from 1 to 7 genes out of 100 downregulated genes (Additional file 6: Table S7).

In summary, the transcriptome of *ΔlitR* relative to the wild-type exhibited an equal gene distribution between upregulated and downregulated genes which suggests that LitR may act both as a positive and negative regulator in *A. salmonicida.*

#### Expression profiling of *A. salmonicidaΔrpoQ* mutant

**Fig. 4:**
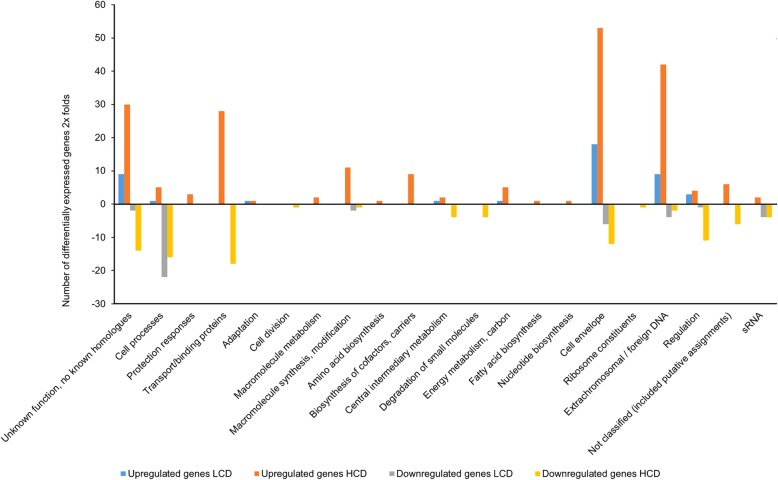
Functional distribution of genes between *A. salmonicida* wild-type and *ΔrpoQ* mutant at LCD and HCD that are ≥2 × differentially expressed

Figure 4, represents the transcriptome of *ΔrpoQ* relative to the wild-type (*ΔrpoQ/*wt) at LCD and HCD. The LCD transcrimtome resulted in a total of 84 DEGs, where 43 (51.2%) were upregulated and 41 (48.8%) were downregulated (Additional file 7: Table S8). At HCD we identified in total 300 DEGs, 206 (68.6%) upregulated and 94 (31.3%) downregulated (Additional file 8: Table S9). The 84 DEGs at LCD were distributed into 8 functional groups (Fig. 4). Among the 43 upregulated genes (LCD), 18 genes (41.8%) were grouped within the *cell envelope* group, where *VSAL_II0252* annotated as hypothetical protein was among the genes with high fold change value (16.1-fold change). Nine genes (20.9%) fell into each of *unknown functions* and *extrachromosomal/foreign DNA*. Three genes (6.9%) were allocated to *regulation* and one gene (*VSAL_II0170*) codes for methyl-accepting chemotaxis protein was grouped in *cell processes* (Additional file 9: Table S10). The 41 downregulated genes were distributed in 7 functional groups with highest number of genes within *cell processes* (*n* = 22, 53.6%). Other downregulated genes fell into *cell envelope* (*n* = 6, 14.6%), *sRNA* and *extrachromosomal/foreign DNA* with 4 genes (9.7%) in each group and *unknown functions* with 2 hypothetical genes *VSAL_I2061* and *VSAL_II1023*.

Figure 4 shows the 300 DEGs at HCD and their distribution among the 21 functional groups. Among the 206 (68.8%) upregulated genes, 53 (25.7%) genes were involved in *cell envelope*, 42 (20%) in *extrachromosomal/foreign DNA* and 30 (14.5%) *hypothetical genes* with unknown functions (Additional file 9: Table S11). The remaining upregulated genes were distributed among other functional groups with a percentage ranging from 13.5 to 0.4% (Additional file 9: Table S11). The 94 downregulated genes at HCD were mostly represented in *transport/binding proteins* (*n* = 18, 19%), *cell processes* (*n* = 16, 17%), *hypothetical proteins* with unknown functions (*n* = 14, 14.8%), *cell envelope* (*n* = 12, 12.7%) and genes involved in *regulation* (*n* = 11, 11.7%). The remaining genes fell into other functional categories and ranging from 1 to 6 genes out of 94 downregulated genes (Additional file 9: Table S11).

The transcriptome of *ΔrpoQ* compared to the wild-type*,* showed more upregulated genes (68.8%), than downregulated (31.2%) at HCD, which indicates that RpoQ acts more as a negative regulator in *A. salmonicida* at high cell density*.*

### Deletion of *litR* and *rpoQ* impacts operons related to quorum sensing

A large number of genes that fell in the *cell processes* functional group in both *ΔlitR* and *ΔrpoQ* were genes involved in the signaling cascade of bacterial chemotaxis and flagellar biosynthesis. Transcriptional analysis of *ΔrpoQ* compared to the wild-type revealed 29 genes that were considerably downregulated at both low and high cell densities. Among the genes that had the greatest transcript abundance at LCD was the gene encoding flagellin A protein, *flaA* (−61.99-fold change)*.* Other flagellin genes were either expressed with lower fold change values such as *flaB* (−2.05-fold change), *flaC* (−6.29-fold change) and *flaE* (−2.70-fold change) or filtered out due to the predetermined criteria for identifying DEGs (fold change value ≥2 and ≤ −2, *p*-value ≤0.05) such as, *flaD* (−1.98-fold change) and *flaF* (−1.8-fold change). In addition to the genes coding for flagellin proteins, genes coding for flagellar basal body rod, ring, hook and cap proteins (*fliD, flaG, flgB-flgL*) showed also reduced level of expression compared to control (wild-type) (Additional file 7: Table S8). Likewise, at HCD 12 out of 16 downregulated genes grouped in *cell processes* were flagellar genes. In particular, the expression of *flaA* was highly decreased with a fold change value of −17.36. The remaining flagellin genes were expressed at a lower level as *flaC* (−2.04-fold change), while others such as *flaB* (−1.4-fold change), *flaD* (−1.4-fold change), *flaE* (−1.6-fold change) and *flaF* (1.17- fold change), were filtered out due to a fold change values below ≤2 and ≥ −2. Genes encoding flagellar basal body rod, ring and hook proteins (from *flgB* to *flgL*) were also downregulated with fold change values ranging from −3.53 to −11.69. In addition to the flagellar genes, 4 genes encoding methyl-accepting chemotaxis proteins were also downregulated such as *VSAL_I2193*, *VSAL_I0799* at LCD, *VSAL_I0712* at HCD and *VSAL_II1022* at both low and high cell densities (Additional file 8: Table S9).

In contrast to *ΔrpoQ* transcriptome (*ΔrpoQ/*wt), the *ΔlitR* transcription profiling (*ΔlitR/*wt) exhibited an increased level of expression among genes involved in cell motility and chemotaxis. One gene, *VSAL_I2117,* encoding methyl-chemotaxis accepting proteins was upregulated with fold change values of 3.84 and 3.46 at low and high cell densities, respectively. Only one flagellin gene, *flaC* gene (*VSAL_I2317*) was found to be upregulated with a fold change of 2.64 at HCD (Additional file 5: Table S5).

**Fig. 5:**
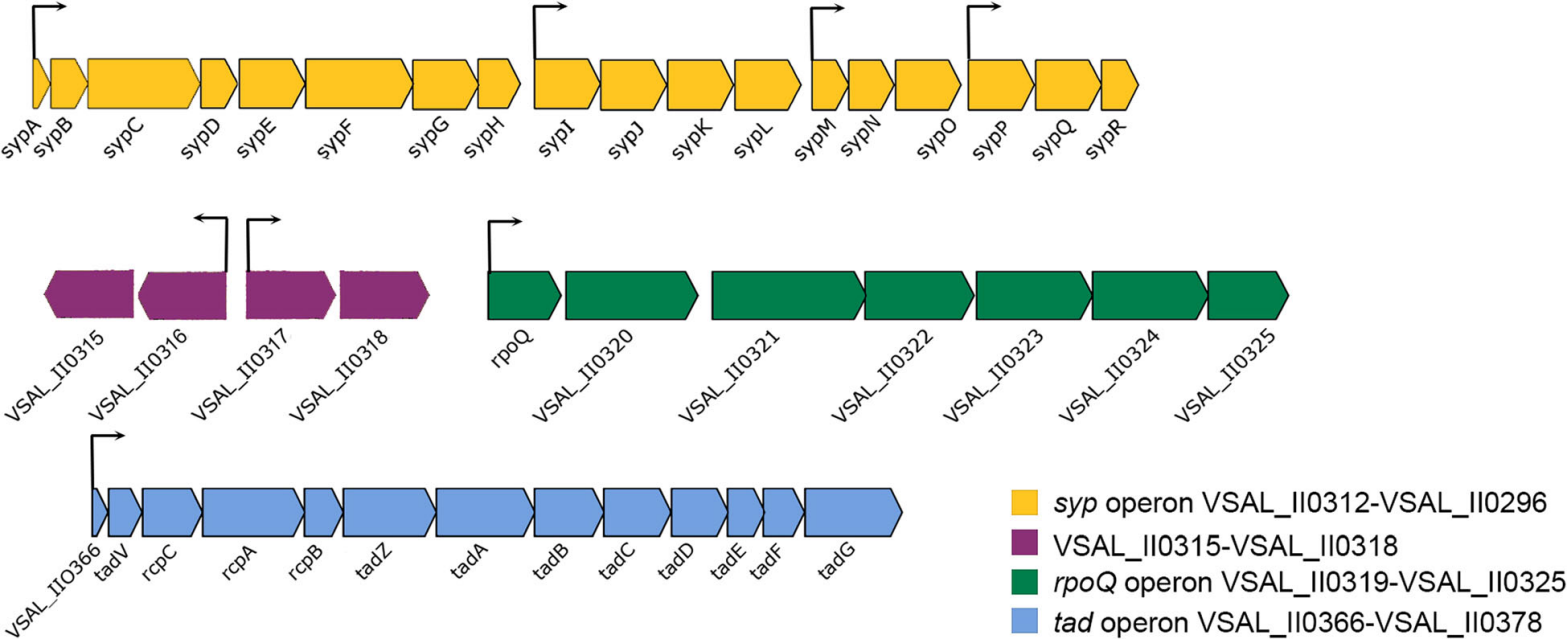
Organization of *syp* genes, *rpoQ* genes and *tad* genes in *A. salmonicida* LFI1238. Arrows indicate genes and their direction of transcription. Color code represents the different operons and their start-end *VSAL* number

The second most highly expressed group of genes are those associated with the *tad* operon. The *tad* operon in *A. salmonicida* consists of 13 genes (*VSAL_II0366* to *VSAL_II0378*) and is located on the second chromosome, that harbours accessory genes [36] (Fig. 5).

The transcriptome of *ΔrpoQ* (*ΔrpoQ/*wt) at LCD showed that all 13 *tad* genes were highly upregulated (Table 3). Nine *tad* genes (*VSAL_II0369*, *VSAL_II0371*, *VSAL_II0372*, *VSAL_II0373*, *VSAL_II0374*, *VSAL_II375*, *VSAL_II0376*, *VSAL_II0377* and *VSAL_II0378*) were classified as *pathogenicity island-related* factors. The other 5 *tad* genes (*VSAL_II0366*, *VSAL_II0367* and *VSAL_II0368*) fell into *surface structures* group coding for Flp-type pilus protein. At HCD 8 out of 13 genes exhibiting an increased level of expression based on our criteria (fold change value ≥2 and ≤ −2, *p*-value ≤0.05). Four *tad* genes were classified within *pathogenicity island*-*related* functions (*VSAL_II0369*, *VSAL_II0371*, *VSAL_II0372* and *VSAL_II0373*), other 4 were divided into *surface structures* (*VSAL_II0366*, *VSAL_II0367*, *VSAL_II0368*) and *membrane exported lipoproteins* (*VSAL_II0370*). All 8 *tad* DEGs ranged from 11.09 to 5.6-fold change (Table 3).

**Table 3:** Genes of the *tad* operon of *ΔlitR*/wt and *ΔrpoQ*/wt at low and high cell densities

VSAL_ID	LCD		HCD		Gene	Function
	FC	p-adjusted	FC	p-adjusted		
*ΔrpoQ*/wt						
* VSAL_II0366*	**25.55**	1.58E-95	**11.88**	1.16E-09		fimbrial protein, Flp/Fap pilin component
* VSAL_II0367*	**24.82**	7.13E-119	**10.45**	8.51E-10	*tadV*	type IV leader peptidase
* VSAL_II0368*	**14.23**	1.86E-123	**6.78**	3.37E-14	*rcpC*	putative Flp pilus assembly protein
* VSAL_II0369*	**10.94**	5.17E-98	**7.24**	4.39E-16	*rcpA*	type II/III secretion system protein
* VSAL_II0370*	**14.26**	3.98E-113	**7.42**	5.14E-10	*rcpB*	putative lipoprotein
* VSAL_II0371*	**13.54**	2.15E-108	**6.16**	NA	*tadZ*	type II secretion system protein Z
* VSAL_II0372*	**12.73**	6.04E-118	**5.63**	1.76E-07	*tadA*	type II/IV secretion system protein, ATP binding domain
* VSAL_II0373*	**10.33**	1.16E-86	**6.99**	4.54E-16	*tadB*	bacterial type II secretion system protein F
* VSAL_II0374*	**4.65**	9.46E-64	1.49	5.18E-01	*tadC*	bacterial type II secretion system protein F
* VSAL_II0375*	**2.94**	1.16E-29	1.18	7.92E-01	*tadD*	putative secretion system protein
* VSAL_II0376*	**3.17**	1.38E-31	1.16	8.00E-01	*tadE*	membrane associated secretion system protein
* VSAL_II0377*	**3.11**	9.37E-30	1.06	9.18E-01	*tadF*	membrane associated secretion system protein
* VSAL_II0378*	**3.04**	1.68E-30	1.11	8.50E-01	*tadG*	membrane associated secretion system protein
*ΔlitR*/wt						
* VSAL_II0366*	**12.23**	7.54E-75	**10.24**	2.94E-13		fimbrial protein, Flp/Fap pilin component
* VSAL_II0367*	**8.59**	3.94E-59	**6.44**	1.20E-12	*tadV*	type IV leader peptidase
* VSAL_II0368*	**4.30**	1.07E-38	**2.42**	NA	*rcpC*	putative Flp pilus assembly protein
* VSAL_II0369*	**3.45**	4.58E-30	**3.10**	6.14E-05	*rcpA*	type II/III secretion system protein
* VSAL_II0370*	**4.67**	1.77E-37	**2.74**	0.009798979	*rcpB*	putative lipoprotein
* VSAL_II0371*	**3.73**	1.31E-24	**2.38**	0.004220895	*tadZ*	type II secretion system protein Z
* VSAL_II0372*	**3.67**	2.53E-27	**2.43**	0.000108788	*tadA*	type II/IV secretion system protein, ATP binding domain
* VSAL_II0373*	**2.49**	1.89E-11	**2.53**	NA	*tadB*	bacterial type II secretion system protein F
* VSAL_II0374*	1.88	7.21E-09	1.06	0.926675356	*tadC*	bacterial type II secretion system protein F
* VSAL_II0375*	1.32	0.031001079	1.29	0.372370396	*tadD*	putative secretion system protein
* VSAL_II0376*	1.45	0.001283239	1.29	0.376863481	*tadE*	membrane associated secretion system protein
* VSAL_II0377*	1.53	0.000365049	1.35	0.33429395	*tadF*	membrane associated secretion system protein
* VSAL_II0378*	1.35	0.006750764	1.35	0.304804669	*tadG*	membrane associated secretion system protein

Values indicated in bold are differentially expressed genes with fold change values (FC) that are ≥2 and ≤ −2, *p*-value ≤0.05

In comparison to *ΔrpoQ*, the *ΔlitR* transcriptome relative to the wild-type revealed fewer *tad* genes to be differentially expressed in our analysis. An equal number of differentially expressed genes was present in both LCD and HCD with approximately similar fold change values (Table 3).

### Exopolysaccharide genes are highly expressed in the *ΔlitR* and *ΔrpoQ* mutants

The inactivation of either *rpoQ* or *litR* in *A. salmonicida* resulted in strains with enhanced extracellular polysaccharide production, which is involved in biofilm formation and wrinkled colony morphology [19, 33] . The biosynthesis of EPS in *A. salmonicida* likely requires the expression of *syp* operon (22,453 bp) located on the second chromosome [36]. The *syp* operon consists of 18 genes (*VSAL_II0295* to *VSAL_II0312*) organized into four transcription units (Fig. 5).

The transcriptome of *ΔrpoQ* compared to the wild-type, showed that 13 *syp* genes were upregulated at HCD, whereas at LCD only *sypB* (*VSAL_II0311*) was differentially expressed with a fold change value of 2.03 (Table 4).

**Table 4:** DEGs of *syp* locus at low and high cell densities in the *ΔrpoQ*/wt

VSAL_ID	LCD		HCD		Gene	Function
	FC	p-adjusted	FC	p-adjusted		
*VSAL_II0295*	1.22	0.74710921	**2.189**	0.07468146	*sypR*	sugar transferase
*VSAL_II0296*	1.07	0.89636775	**2.585**	0.00426114	*sypQ*	putative transmembrane glycosyl transferase
*VSAL_II0297*	1.10	0.84634234	**3.182**	0.0001044	*sypP*	putative glycosyl transferase
*VSAL_II0298*	−1.05	0.91873517	**2.462**	0.04236082	*sypO*	putative membrane protein
*VSAL_II0299*	1.14	0.81791828	**2.189**	0.11185209	*sypN*	putative glycosyl transferases
*VSAL_II0300*	1.43	0.17621734	**3.160**	0.00642603	*sypM*	hypothetical protein
*VSAL_II0301*	−1.25	0.56460157	1.778	0.44063722	*sypL*	O-antigen polymerase
*VSAL_II0302*	1.12	0.85566635	**2.713**	0.04069669	*sypK*	putative polysaccharide biosynthesis protein
*VSAL_II0303*	1.10	0.87199934	**2.928**	0.00991193	*sypJ*	putative glycosyl transferase
*VSAL_II0304*	1.23	0.60244622	**2.868**	0.00187435	*sypI*	putative glycosyl transferase
*VSAL_II0305*	−1.23	0.602336	1.035	0.96181635	*sypH*	putative glycosyl transferase
*VSAL_II0306*	−1.35	0.2022748	−1.647	0.06439411	*sypG*	two-component response regulator, transcriptional regulatory protein LuxO
*VSAL_II0307*	−1.17	0.69188251	−1.157	0.76925525	*sypF*	response regulator, histidine kinase
*VSAL_II0308*	1.25	0.4506543	1.021	0.97005546	*sypE*	putative response regulator
*VSAL_II0309*	1.20	0.73767376	**2.189**	0.08945561	*sypD*	putative capsular polysaccharide synthesis protein
*VSAL_II0310*	1.49	0.08145483	**3.811**	0.00016578	*sypC*	polysaccharide biosynthesis/export protein
*VSAL_II0311*	**2.03**	0.00119744	**4.377**	0.00012723	*sypB*	outer membrane protein, OmpA family
*VSAL_II0312*	1.94	0.01339391	**6.063**	5.41E-06	*sypA*	hypothetical protein, putative anti-sigma factor antagonist

Values indicated in bold are differentially expressed genes with fold change values (FC) that are ≥2 and ≤ −2, p-value ≤0.05

Next, we wanted to analyze the importance of *syp* genes in formation of colony rugosity and biofilm and for this 3 *syp* genes (*sypQ, sypP* and *sypC*) were separately inactivated in the wild-type LFI1238 and *ΔrpoQ* mutant by insertional inactivation. The constructed mutants were GFP tagged for better biofilm visualization. The inactivation of *sypQ, P* or *C* in *ΔrpoQ* resulted in strains similar to the wild-type strain with no biofilm formation and smooth colonies (Additional file 10: Figure S1). No difference was observed on biofilm formation or colony morphology after the inactivation of *syp* genes in *A. salmonicida* wild-type at the chosen conditions (Additional file 10: Figure S1).

The transcriptome of *ΔlitR* (*ΔlitR/*wt) did not show any significant upregulation of the *syp* genes, except for two genes; *sypA* (*VSAL_II0312*) and *sypC* (*VSAL_II0310*) encoding a putative anti-sigma factor and polysaccharide biosynthesis/export protein, respectively (Additional file 5: Table S5). Our results indicate that this operon is regulated in a cell density dependent manner, where RpoQ expression leads to a repression of large number of *syp* genes at HCD.

### Comparative analysis of *ΔrpoQ* and *ΔlitR* reveals genes regulated by QS

**Fig. 6:**
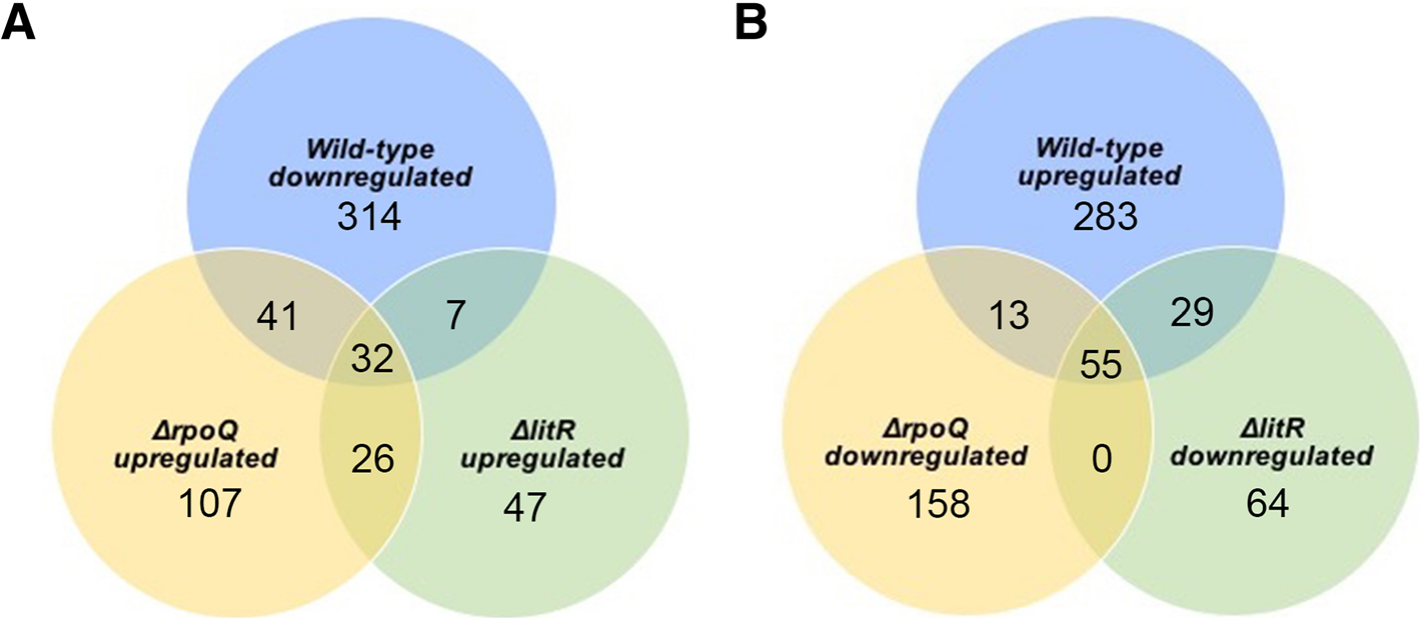
Venn diagram of differentially expressed genes **a**) Venn diagram of upregulated genes in the *ΔlitR* and *ΔrpoQ* mutants and downregulated genes in the wild-type at HCD*.* **b**) Venn diagram of downregulated genes in the *ΔlitR* and *ΔrpoQ* mutants and upregulated genes in the wild-type at HCD*.* The sum of the numbers in each large circle represents total number of uniquely differentially expressed genes identified in each sample. The overlap part of the circles represents number of overlapping differentially expressed genes between combinations

RpoQ and LitR were studied previously and shown to regulate phenotypes such as motility, adhesion, biofilm formation and colony morphology differently in *A. salmonicida* [19, 33]. To identify genes that are differentially expressed in *ΔrpoQ* relative to *ΔlitR,* we compared the RNA-Seq data for these mutants at low and high cell densities using DESeq. At LCD a differential expression analysis revealed 63 (53.3%) and 55 (46.6%) of the total 118 genes to be significantly up and downregulated respectively (Additional file 11: Table S12). Whereas at HCD the RNA-Seq revealed 107 genes where 55 (51.4%) were upregulated while 57 (53.2%) were downregulated. Figure 6 illustrates the number of DEGs that overlap between the *ΔrpoQ* and *ΔlitR* transcriptome where the majority of the differentially expressed genes at both cell densities came from chromosome I. At both low and high cell densities, genes associated with several phenotypes known to be related to QS were significantly expressed in the *ΔrpoQ* relative to *ΔlitR.* Among these were genes involved in motility and chemotaxis, genes associated with the *syp* operon such as (*VSAL_II0297*) encoding a putative glycosyl transferase, (*VSAL_II0300*) annotated as hypothetical protein, (*VASL_II0311*) coding for the outer membrane protein OmpA and (*VSAL_II0312*) coding for a putative anti-sigma factor, in addition to some genes associated with the *tad* operon (Additional file 11: Table S12 and Additional file 12: Table S13).

## Discussion

Whole-transcriptome RNA sequencing analysis provides a powerful understanding of the gene expression patterns underlying the basic biology of the organism. In this work we studied the comparative transcriptome of *A. salmonicida* LFI1238, *ΔlitR* and *ΔrpoQ* mutants at low (OD_600_ = 0.3) and high (OD_600_ = 1.2) cell densities in SWT medium at 8°C. The SWT medium (2.5% salt concentration) and low temperature (8°C) were chosen as appropriated physiological conditions (similar to ocean environment) for *A. salmonicida* which is responsible for developing of cold-water vibriosis in Atlantic salmon at low seawater temperatures [39–41]. These conditions also favoured the development of several phenotypes (as motility, morphology and biofilm) related to QS in our *ΔlitR* and *ΔrpoQ* mutants in vitro [19, 33]. The differentially expressed genes identified in this work provide a new insight to explain mechanisms related to QS such as motility, bioluminescence, wrinkled colony morphology, adhesiveness and biofilm formation.

### Changes in cell density impacts genes related to quorum sensing in *A. salmonicida* LFI1238

QS is known to be a cell density dependent mechanism allowing communication between bacteria and is regulated through master regulators, as VanT, HapR and LitR [28, 42, 43]. LitR was shown previously to regulate cryptic bioluminescence in *A. salmonicida*, where its inactivation resulted in less light production [44]. This led us to propose that cryptic bioluminescence is a high cell density dependen phenotype, where LitR is involved in its regulation. Herein, the transcriptome of *A. salmonicida* at HCD showed a significant upregulation of *lux* operon (Additional file 2: Table S2), confirming that the alteration in gene experession of this operon is affected by changes in population.

RpoS sigma factor aids in adaptation to environmental stress, mainly required for virulence, stress resistance and biofilm formation, additionally it has been shown to be required for full motility in some vibrios [45]. In this study *rpoQ* (RpoS-like sigma factor) was found to be upregulated in *A. salmonicida* at HCD compared to LCD. Moreover, the transcriptome of *A. salmonicida* demonstrated a downregulation in genes associated with motility and chemotaxis. This explains our previously obtained results, where the overexpression of RpoQ in the wild-type resulted in non-motile strains [33]. Hence, the expression of *rpoQ* leads to reduced motility in *A. salmonicida* at HCD. *So why do A. salmonicida reduce their motility at HCD?* It is believed that bacteria have different expression profiles during the different stages of life cycle. However, a complete life cycle of *A. salmonicida* is still unknown. But we assume that *A. salmonicida* similar to *V. cholerae*, is able to change from planktonic to biofilm life cycle which results in changes in genes expression required for motility and other functions [46, 47]. The high cell density transcriptome presented in this study exhibits the activities of the late exponential phase (OD_600_ = 1.2). During this phase nutrition accessibility is limited which favors the bacterial cells to enter the stationary phase and QS. Thus, at this time period the accumulation of autoinducers results in the expression of LitR, which in turn activates the *rpoQ* expression leading to activate regulators responsible for motility reduction, hence protecting the bacteria from excessive energy loss required to manage the motility apparatus. Additionally, it has been shown that *A. salmonicida* suppresses motility under the late stages of the host colonization (i.e., HCD) [48, 49]. In contrast to HCD, at LCD we believe that the expression of motility genes in *A. salmonicida* are upregulated resulting in motile strains able to swim and colonize new host or environment. However the mechanism by which flagellar biosynthesis is controlled in *A. salmonicida* seems to be complex and will require further studies.

### LitR and RpoQ regulate genes vital for motility

*A. salmonicida* is motile by nine polar flagella [50], where genes required for flagellum biosynthesis and flagellar motility are organized in different loci (Fig. 2) in a similar manner to *A. fischeri* [49]. The expression of genes involved in the synthesis of flagella in vibrios is tightly regulated through a complex hierarchy requiring the presence of regulatory proteins and the production of the flagellin monomer the basic component of bacterial flagellum, such as, FlaA [10, 51]. RpoQ was shown to be a positive regulator of motility in *A. salmonicida* under our experimental conditions [33], and here we determine that the deletion of *rpoQ* resulted in a downregulation of several flagellar and chemotaxis genes, mainly *flaA* at both cell densities. Although *A. salmonicida* flagellar filament is composed of six flagellins (Fig. 2), it appears that the FlaA protein is mainly essential for motility and most likley regulated by RpoQ. The importance of FlaA for motility was reported in *V. cholerae,* where its deletion affected motility and thereby virulence [44]. Similarly, in *A. fischeri* the inactivation of *flaA* resulted in strains with reduced motility and symbiotic competence [52]. Likewise, a considerable importance of FlaA for motility was recently documented in *A. salmonicida* LFI1238, where the complete deletion of *flaA* resulted in 62% reduced motility at 8°C [53]. A similar reduction in motility was observed for the *ΔrpoQ* using the same temperature and salt concentration [33]. RpoQ is similar to other sigma factors that functions as a gene activator, and most probably activates a regulator of *flaA* gene*.* In *V. cholerae* it was show that *flaA* transcription is regulated by sigma factor 54 which depends on and requires an additional regulator, FlrC [54, 55]. Thus, it is reasonable to speculate that RpoQ may work in the similar manner as *V. cholerae* by activating regulators responsible for motility, where in the *ΔrpoQ* mutant, *flaA* regulator is not activated resulting in decreased motility.

The quorum sensing master regulator LitR, has been shown to be associated with motility in *A. salmonicida* similar to other bacteria [27, 56]. The deletion of *litR* (*ΔlitR*) resulted in more motile strain than the wild-type [27]. This led us to conclude that LitR is a repressor of motility at HCD, where its deletion (*ΔlitR*) mimics the low cell density phenotype [27]. A similar conclusion was also applied to the role of RpoQ in motility [33]. However, *ΔrpoQ* transcriptome exhibited downregulation in motility genes regardless of growth phases. This proposes either that QS does not seem to be implicated in the RpoQ-dependent induction of motility and chemotaxis, or that *rpoQ* is critical for flagellar gene expression, where its deletion does not completely mimic the low cell density phenotype.

In summary, these results indicate the importance of RpoQ in controlling the *flaA* gene which has a direct impact on the motility. Additionally RpoQ seems to tightly regulates several genes essential for flagellar assembly of *A. salmonicida*. Furthermore, RpoQ is believed to be a stress regulator in *A. salmonicida* similar to RpoS which may have the ability to switch between motile and non-motile states in response to physical or chemical changes in the environment.

### LitR and RpoQ repress genes associated with virulence

Among the differentially expressed transcripts of *ΔrpoQ* and *ΔlitR* we were able to identify a number of significantly upregulated genes that may play an important role in virulence. These included the genes encoding adhesion and fimbrial attachment proteins also known as *tad* genes or *tad* operon. Tad loci is a widespread colonization island that is found in numerous pathogenic and non pathogenic bacteria including vibrios such as *V. cholerae*, *A. fischeri, V.vulnificus* and *Vibrio parahaemolyticus* (*V. parahaemolyticus*) [36, 57]. The *A. salmonicida* genome encode a number of potential virulence factors. Among them is the Flp-type pilus (fimbrial –low molecular weight protein), which has high similarity to the Tad macromolecular transport system of *Actinobacillus actinomycetemcomitans* (*A. actinomycetemcomitans*) [36]. Tad operon is known to facilitate adhesion and, to play an important role in motility and biofilm formation [57]. Although the function of the *tad* operon was not investigated in detail in *A. salmonicida* and the inactivation of two *tad* genes (*VSAL_II0367* and *VSAL_II0368*) did not affect the architecture or amounts of biofilm formed [19], it is reasonable to assume that this widespread colonization island provides important functions for pathogenic bacteria (e.g., *A. salmonicida*) in the form of colonization and adhesion. Our previous microarray analyzes on the *ΔlitR* mutant did not reveal any *tad* genes to be differentially expressed [19], although the adhesion of the *ΔlitR* mutants to the agar plates was observed [27]. In the study presented here, DEGs related to Tad locus in *ΔrpoQ* and *ΔlitR* yielded highly similar findings, where a number of *tad* genes were significantly upregulated. Whereas, the transcriptome of *A. salmonicida* wild-type at HCD revealed opposite results, where *tad* genes were downregulated. Thus, the increased expression level of LitR and RpoQ at HCD, leads to a repression of *tad* genes in *A. salmonicida* wild-type. This, proposes the importance of this colonization island at early stages of life cycle (i.e., LCD). Although evidence for the physiological role of this colonization island in *Vibrionaceae* is scant, recently a correlation between *tad* genes and phenotypes in *V. vulnificus* was found to be associated with biofilm formation, auto-aggregation and initial surface attachment to the host [58]. *tad* genes were also found to mediate adherence, colonization and micro-colony formation in other bacteria [59–61]. Hypothetically, these findings also can be considered in *A. salmonicida,* where the *tad* operon is mainly required for the initial surface attachment of the cells to the biotic surface and formation of micro-colonies and less necessary in the later stages of biofilm or infection. However, further investigations are needed to confirm this hypothesis.

### Biofilm formation and colony rugosity are low cell density phenotypes involving expression of *syp*

**Fig. 7:**
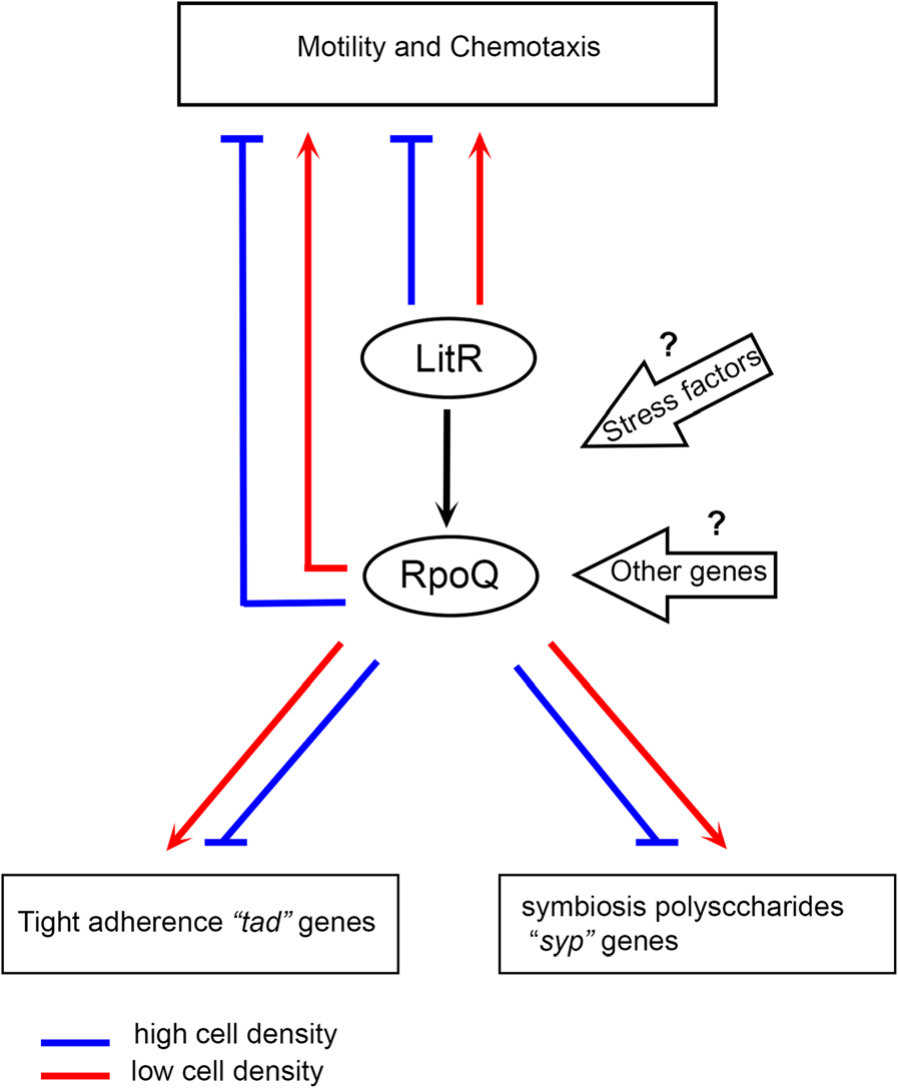
Proposed model of QS and the possible LitR and RpoQ interaction in *A. salmonicida.* The expression of LitR signaling at high cell density represses motility, biofilm and activates transcription of RpoQ [19, 27]. The increased level of RpoQ activity leads to strong repression on biofilm formation, rugose colony morphology, motility and adhesion, through a negative regulatory cascade on EPS producing genes (i.e., *syp*), flagellar and *tad* genes, respectively. At low cell density the LitR is not activated, thereby RpoQ levels are low and not sufficient to repress either *tad* or *syp* genes, resulting in an upregulation leading to a strong adhesion to surface and thereby biofilm formation. However, the deletion of *rpoQ* results in reduced motility, where the regulation of flagellar genes maybe affected by other genes and environmental factor either dependent or independent of QS mechanism. Arrows and lines with bar end indicate positive and negative regulation respectively. Lines may also indirect direct or indicate pathways with several steps

The ability to form rugose colonies and biofilm are often correlated features in vibrios, which is generally associated with enhanced production of exopolysaccharides [21, 25, 62]. Similarly, in *A. salmonicida* colony rugosity and biofilm formation requires the expression of *syp* genes responsible for the production of EPS [19, 33]. Our previous microarray analysis showed that the expression of 14 out of 18 *syp* genes was negatively regulated by LitR, where the majority, were genes significantly upregulated in the biofilm compared to the suspension [19]. However, the data obtained from the current work did not show significant upregulation of the 14 *syp* genes previously identified [19], except *sypA* and *sypC* genes, that showed to be differentially expressed at HCD. We know from our previous results that changes in medium composition affects the biofilm morphology [19], and here we assume that changes in some compounds of the SWT medium have affected the transcriptome of *ΔlitR* and resulted in less differentially expressed *syp* genes. In contrast to the *ΔlitR* transcriptome, the *ΔrpoQ* presented an upregulation among 13 out of 18 *syp* genes at HCD. We have previously observed what we refere to as a “late and weak” wrinkling colony morphology exhibited by *ΔlitR* compared to *ΔrpoQ,* which demonstrated an earlier and stronger rugosity in addition to a heavy and slimy extracellular matrix substance in the biofilm [33]. This led us to propose that LitR performs its activity on *syp* through RpoQ, where its expression leads to a strong *syp* repression. Moreover, the mature biofilm formation exhibited by *ΔlitR* was proposed to be a result of two independent processes where the first results in repression of *syp* via RpoQ while the second is independent of *rpoQ* and represses other biofilm matrix components. When three *syp* genes were inactivated separately in the *rpoQ* mutant no biofilm and no wrinckeled colonies were formed, and the *ΔrpoQsyp* double mutants behaved similar to the wild-type (Additional file 10: Figure S1). However, the inactivation of the same *syp* genes in *ΔlitR*, resulted in some biofilm production using the same conditions [19]. Hence, the inactivation of *syp* genes in *ΔrpoQ* mutant inhibited colony rugosity and biofilm formation completely, which was not the case for the *ΔlitR*. Consequently, our results provide a clear evidence that the negative regulatory cascade from LitR to *syp* genes is operated through RpoQ in a cell density dependent manner. *Why is RpoQ involved in regulating exopolysaccharide production* *via* *syp?* The bacteria, whether it is in the host or in the aquatic environment, employes survival strategies, where sigma factors (e.g., RpoS or RpoQ) are believed to aid in adaptation to environmental stress such as osmotic shock and starvation [63]. Hence, for RpoQ to be involved in regulating this EPS locus (*syp* operon) may suggest that this sigma factor may play an important role in environmental persistence protecting the bacteria under starvation and during infection of the host. We therefore believe that in addition to the negative regulatory cascade operated from LitR to *syp* genes (via RpoQ), *rpoQ* is also influenced by other genes and environmental factors leading to repression of *syp* in a pathway that remains unknown (Fig. 7).

Even though the relationship between RpoQ and LitR is not well-studied in *A. salmonicida,* our current transcriptome and previous microarray data showed a positive regulation of LitR on *rpoQ*, confirming that RpoQ operates downstream of LitR in the QS regulatory hierarchy [19]. Furthermore, the overexpression of *rpoQ* in the *ΔlitR* mutant influenced phenotypes related to QS [33]. Consistent with the results demonstrated in *A. fischeri*, where the overexpression of RpoQ in *ΔlitR* mutant resulted in decreased motility [32].

Taken together, our data suggest a working model (Fig. 7) for *how LitR and RpoQ work together in A. salmonicida*, proposing that expression of genes in *A. salmonicida* is not always regulated by QS, and possibly involve other regulatory elements that act independently of the QS regulatory mechanism. Hence, the interaction between RpoQ and LitR and their roles in controlling motility, biofilm formation and rugose colony morphology, may be directly or indirectly regulated by RpoQ independent of LitR and vice versa. Additionally, we assume that RpoQ is regulated by other gene(s) and stress factors rather than LitR alone.

## Conclusion

In this work we have shown that the master regulator LitR and the alternative sigma factor RpoQ regulate genes involved in motility, rugose colony morphology and biofilm formation in *A. salmonicida*. Our results indicate that RpoQ is an activator of *flaA* gene either directly or indirectly. Moreover, the positive activation of LitR on *rpoQ* results in reduced motility, repression of genes involved in adhesion (e.g., *tad* genes) and exopolysaccharide production via *syp* operon at HCD in *A. salmonicida* wild-type. These findings confirm that LitR and RpoQ regulate phenotypic traits related to QS together (dependent) and also independent of each other, where other environmental factors and genes are probably also involved. However further studies are needed to map the elements and factors affecting gene expression and influencing the observed phenotypes during different life cycles.

## Additional files

Additional file 1: **Table S1.** The table lists the summary of RNA sequencing data for *A. salmonicida* LFI1238, *ΔlitR* and *ΔrpoQ*. (XLSX 10 kb)

Additional file 2: **Table S2.** The table lists the differentially expressed genes of *A. salmonicida* wild-type at HCD compared to LCD. (XLSX 82 kb)

Additional file 3: **Table S3.** The table lists the functional distribution of the differenatially expressed gene of *A. salmonicida* at HCD relative to LCD. (DOCX 16 kb)

Additional file 4: **Table S4.** The table lists the differentially expressed genes of *ΔlitR* mutant compared to wild-type at LCD. (XLSX 13 kb)

Additional file 5: **Table S5.** The table lists the differentially expressed genes of *ΔlitR* mutant compared to wild-type at HCD. (XLSX 24 kb)

Additional file 6: **Table S6** and **Table S7.** The tables list the functional distribution of *ΔlitR*/wt at LCD and HCD. (DOCX 18 kb)

Additional file 7: **Table S8.** The table lists the differentially expressed genes of *ΔrpoQ* mutant compared to wild-type at LCD. (XLSX 15 kb)

Additional file 8: **Table S9.** The table lists the differentially expressed genes of *ΔrpoQ* mutant compared to wild-type at HCD. (XLSX 28 kb)

Additional file 9: **Table S10** and **Table S11.** The tables list the functional distribution of *ΔrpoQ*/wt at LCD and HCD. (DOCX 18 kb)

Additional file 10: **Figure S1.** Colony morpgology and biofilm formation of *ΔrpoQ* and LFI1238 *syp* mutants. (DOCX 1263 kb)

Additional file 11: **Table S12.** The table lists the differentially expressed genes of *ΔrpoQ* compared to *ΔlitR* at LCD. (XLSX 19 kb)

Additional file 12: **Table S13.** The table lists the differentially expressed genes of *ΔrpoQ* compared to *ΔlitR* at HCD. (XLSX 17 kb)

## Abbreviations

DEGs: Differentially expressed genes
EPS: Exopolysaccharide
HCD: High cell density
LCD: Low cell density
min: Minutes
OD_600_: Optical density measured at 600 nm
ON: Overnight
QS: Quorum sensing
rpm: Rounds per minute
