# Supplementary material for: Differential expression profiling of ΔlitR and ΔrpoQ mutants reveals insight into QS regulation of motility, adhesion and biofilm formation in Aliivibrio salmonicida
Source: BMC Genomics. 2019 Mar 15;20:220. doi: 10.1186/s12864-019-5594-4 (PMC6420764; doi:10.1186/s12864-019-5594-4)
Supplement: Supplementary file 3 — Table S3. The table lists the functional distribution of the differenatially expressed gene of A. salmonicida at HCD relative to LCD. (DOCX 16 kb) [file 12864_2019_5594_MOESM3_ESM.docx]

**Additional file 3**

**Table S3 The functional distribution of one thousand and thirteen DEGs of wt1.2/wt0.3** The table represents the number of up (*n* = 597) and downregulated (*n* = 416) genes with their percentage distribution within the different functional groups.

| **Functional categories** | **Upregulated genes (n=597)** | | **Downregulated genes (n=416)** | |
| --- | --- | --- | --- | --- |
|  | *Number of genes (n)* | *Percentage (%)* | *Number of genes (n)* | *Percentage (%)* |
| *Unknown function, no known homologues* | 94 | 15.7 | 40 | 9.6 |
| *Cell processes* | 4 | 0.6 | 37 | 8.8 |
| *Protection responses* | 3 | 0.5 | 3 | 0.7 |
| *Transport/binding proteins* | 73 | 12.2 | 57 | 13.7 |
| *Adaptation* | 8 | 1.3 | 2 | 0.4 |
| *Cell division* | 3 | 0.5 | 1 | 0.2 |
| *Macromolecule metabolism* | 20 | 3.3 | 6 | 1.4 |
| *Macromolecule synthesis, modification* | 13 | 2.1 | 21 | 5.0 |
| *Amino acid biosynthesis* | 14 | 2.1 | 3 | 0.7 |
| *Biosynthesis of cofactors, carriers* | 8 | 1.3 | 11 | 2.6 |
| *Central intermediary metabolism* | 29 | 4.8 | 6 | 1.4 |
| *Degradation of small molecules* | 29 | 4.8 | 9 | 2.1 |
| *Energy metabolism, carbon* | 34 | 5.6 | 11 | 2.6 |
| *Fatty acid biosynthesis* | 3 | 0.5 | 0 | 0 |
| *Nucleotide biosynthesis* | 0 | 0 | 8 | 1.9 |
| *Cell envelope* | 97 | 16.2 | 57 | 13.7 |
| *Ribosome constituents* | 2 | 0.3 | 47 | 11.2 |
| *Extrachromosomal / foreign DNA* | 28 | 4.6 | 48 | 11.5 |
| *Regulation* | 59 | 9.8 | 8 | 1.9 |
| *Not classified (included putative assignments)* | 31 | 5.1 | 8 | 1.9 |
| *sRNA* | 45 | 7.5 | 33 | 7.9 |
