# Supplementary material for: Differential expression profiling of ΔlitR and ΔrpoQ mutants reveals insight into QS regulation of motility, adhesion and biofilm formation in Aliivibrio salmonicida
Source: BMC Genomics. 2019 Mar 15;20:220. doi: 10.1186/s12864-019-5594-4 (PMC6420764; doi:10.1186/s12864-019-5594-4)
Supplement: Supplementary file 6 — Table S6 and Table S7. The tables list the functional distribution of ΔlitR/wt at LCD and HCD. (DOCX 18 kb) [file 12864_2019_5594_MOESM6_ESM.docx]

**Additional file 6**

**Table S6 The functional distribution of sixty-two DEGs of *ΔlitR*/wt at LCD**. The table represents the number of up- (*n* = 31) and downregulated (*n* = 31) genes with their percentage distribution within the different functional groups.

| **Functional categories** | **Upregulated genes (n=31)** | | **Downregulated genes (n=31)** | |
| --- | --- | --- | --- | --- |
|  | *Number of genes (n)* | *Percentage (%)* | *Number of genes (n)* | *Percentage (%)* |
| *Unknown function, no known homologues* | 5 | 16.1 | 7 | 16.1 |
| *Cell processes* | 1 | 3.2 | 0 | 0 |
| *Transport/binding proteins* | 5 | 16.1 | 3 | 9.6 |
| *Macromolecule metabolism* | 1 | 3.2 | 1 | 3.2 |
| *Macromolecule synthesis, modification* | 0 | 0 | 1 | 3.2 |
| *Amino acid biosynthesis* | 0 | 0 | 1 | 3.2 |
| *Biosynthesis of cofactors, carriers* | 0 | 0 | 2 | 6.4 |
| *Central intermediary metabolism* | 0 | 0 | 1 | 3.2 |
| *Fatty acid biosynthesis* | 0 | 0 | 1 | 3.2 |
| *Cell envelope* | 10 | 32.2 | 5 | 16.1 |
| *Extrachromosomal / foreign DNA* | 5 | 16.1 | 3 | 9.6 |
| *Regulation* | 1 | 3.2 | 4 | 12.9 |
| *Not classified (included putative assignments)* | 2 | 6.4 | 1 | 3.2 |
| *sRNA* | 1 | 3.2 | 1 | 3.2 |

**Table S7 The functional distribution of two-hundred and twelve DEGs of *ΔlitR*/wt at HCD**. The table represents the number of up (*n* = 112) and down regulated (*n* = 100) genes with the percentage of transcripts within the different functional groups.

| **Functional categories** | **Upregulated genes (*n=*112)** | | **Downregulated genes (*n*=100)** | |
| --- | --- | --- | --- | --- |
|  | *Number of genes (n)* | *Percentage (%)* | *Number of genes (n)* | *Percentage (%)* |
| *Unknown function, no known homologues* | 9 | 8.0 | 19 | 19 |
| *Cell processes* | 2 | 1.7 | 0 | 0 |
| *Protection responses* | 0 | 0 | 1 | 1 |
| *Transport/binding proteins* | 23 | 20.5 | 18 | 18 |
| *Adaptation* | 0 | 0 | 1 | 1 |
| *Cell division* | 0 | 0 | 1 | 1 |
| *Macromolecule metabolism* | 2 | 1.7 | 5 | 5 |
| *Macromolecule synthesis, modification* | 2 | 1.7 | 5 | 5 |
| *Amino acid biosynthesis* | 1 | 0.8 | 0 | 0 |
| *Biosynthesis of cofactors, carriers* | 2 | 1.7 | 0 | 0 |
| *Central intermediary metabolism* | 5 | 4.4 | 2 | 2 |
| *Energy metabolism, carbon* | 6 | 5.3 | 1 | 1 |
| *Fatty acid biosynthesis* | 0 | 0 | 2 | 2 |
| *Cell envelope* | 17 | 15.1 | 19 | 19 |
| *Ribosome constituents* | 0 | 0 | 1 | 1 |
| *Extrachromosomal / foreign DNA* | 28 | 25 | 1 | 1 |
| *Regulation* | 6 | 5.3 | 12 | 12 |
| *Not classified (included putative assignments)* | 7 | 6.2 | 7 | 7 |
| *sRNA* | 2 | 1.7 | 3 | 3 |
