# Supplementary material for: Differential expression profiling of ΔlitR and ΔrpoQ mutants reveals insight into QS regulation of motility, adhesion and biofilm formation in Aliivibrio salmonicida
Source: BMC Genomics. 2019 Mar 15;20:220. doi: 10.1186/s12864-019-5594-4 (PMC6420764; doi:10.1186/s12864-019-5594-4)
Supplement: Supplementary file 9 — Table S10 and Table S11. The tables list the functional distribution of ΔrpoQ/wt at LCD and HCD. (DOCX 18 kb) [file 12864_2019_5594_MOESM9_ESM.docx]

**Additional file 9**

**Table S10 The functional distribution of eighty four DEGs of *ΔrpoQ*/wt at LCD**. The table represents the number of up (*n* = 43) and downregulated (*n* = 41) genes with their percentage distribution within the different functional groups

| **Functional categories** | **Upregulated genes (n=43)** | | **Downregulated genes (n=41)** | |
| --- | --- | --- | --- | --- |
|  | *Number of genes (n)* | *Percentage (%)* | *Number of genes (n)* | *Percentage (%)* |
| *Unknown function, no known homologues* | 9 | 20.9 | 2 | 4.8 |
| *Cell processes* | 1 | 2.3 | 22 | 53.6 |
| *Adaptation* | 1 | 2.3 | 0 | 0 |
| *Macromolecule synthesis, modification* | 0 | 0 | 2 | 4.8 |
| *Cell envelope* | 18 | 41.8 | 6 | 14.6 |
| *Extrachromosomal / foreign DNA* | 9 | 20.9 | 4 | 9.7 |
| *Regulation* | 3 | 6.9 | 1 | 2.4 |
| *sRNA* | 0 | 0 | 4 | 9.7 |

**Table S11 The functional distribution of three hundred DEGs of *ΔrpoQ*/wt at HCD**. The table represents the number of up (*n* = 206) and down regulated (*n* = 94) genes with their percentage distribution within the different functional groups.

| **Functional categories** | **Upregulated genes (n=206)** | | **Downregulated genes (n=94)** | |
| --- | --- | --- | --- | --- |
|  | *Number of genes (n)* | *Percentage (%)* | *Number of genes (n)* | *Percentage (%)* |
| *Unknown function, no known homologues* | 30 | 14.5 | 14 | 14.8 |
| *Cell processes* | 5 | 2.4 | 16 | 17 |
| *Protection responses* | 3 | 1.4 | 0 | 0 |
| *Transport/binding proteins* | 28 | 13.5 | 18 | 19 |
| *Adaptation* | 1 | 0.4 | 0 | 0 |
| *Cell division* | 0 | 0 | 1 | 1 |
| *Macromolecule metabolism* | 2 | 0.9 | 0 | 0 |
| *Macromolecule synthesis, modification* | 11 | 5.0 | 1 | 1 |
| *Amino acid biosynthesis* | 1 | 0.4 | 0 | 0 |
| *Biosynthesis of cofactors, carriers* | 9 | 4.3 | 0 | 0 |
| *Central intermediary metabolism* | 2 | 0.9 | 4 | 4.2 |
| *Degradation of small molecules* | 0 | 0 | 4 | 4.2 |
| *Energy metabolism, carbon* | 5 | 2.4 | 0 | 0 |
| *Fatty acid biosynthesis* | 1 | 0.4 | 0 | 0 |
| *Nucleotide biosynthesis* | 1 | 0.4 | 0 | 0 |
| *Cell envelope* | 53 | 25.7 | 12 | 12.7 |
| *Ribosome constituents* | 0 | 0 | 1 | 1 |
| *Extrachromosomal / foreign DNA* | 42 | 20 | 2 | 0 |
| *Regulation* | 4 | 1.9 | 11 | 11.7 |
| *Not classified (included putative assignments)* | 6 | 2.9 | 6 | 6.3 |
| *sRNA* | 2 | 0.9 | 4 | 4.2 |
