# Supplementary material for: Differential expression profiling of ΔlitR and ΔrpoQ mutants reveals insight into QS regulation of motility, adhesion and biofilm formation in Aliivibrio salmonicida
Source: BMC Genomics. 2019 Mar 15;20:220. doi: 10.1186/s12864-019-5594-4 (PMC6420764; doi:10.1186/s12864-019-5594-4)
Supplement: Supplementary file 10 — Figure S1. Colony morpgology and biofilm formation of ΔrpoQ and LFI1238 syp mutants. (DOCX 1263 kb) [file 12864_2019_5594_MOESM10_ESM.docx]

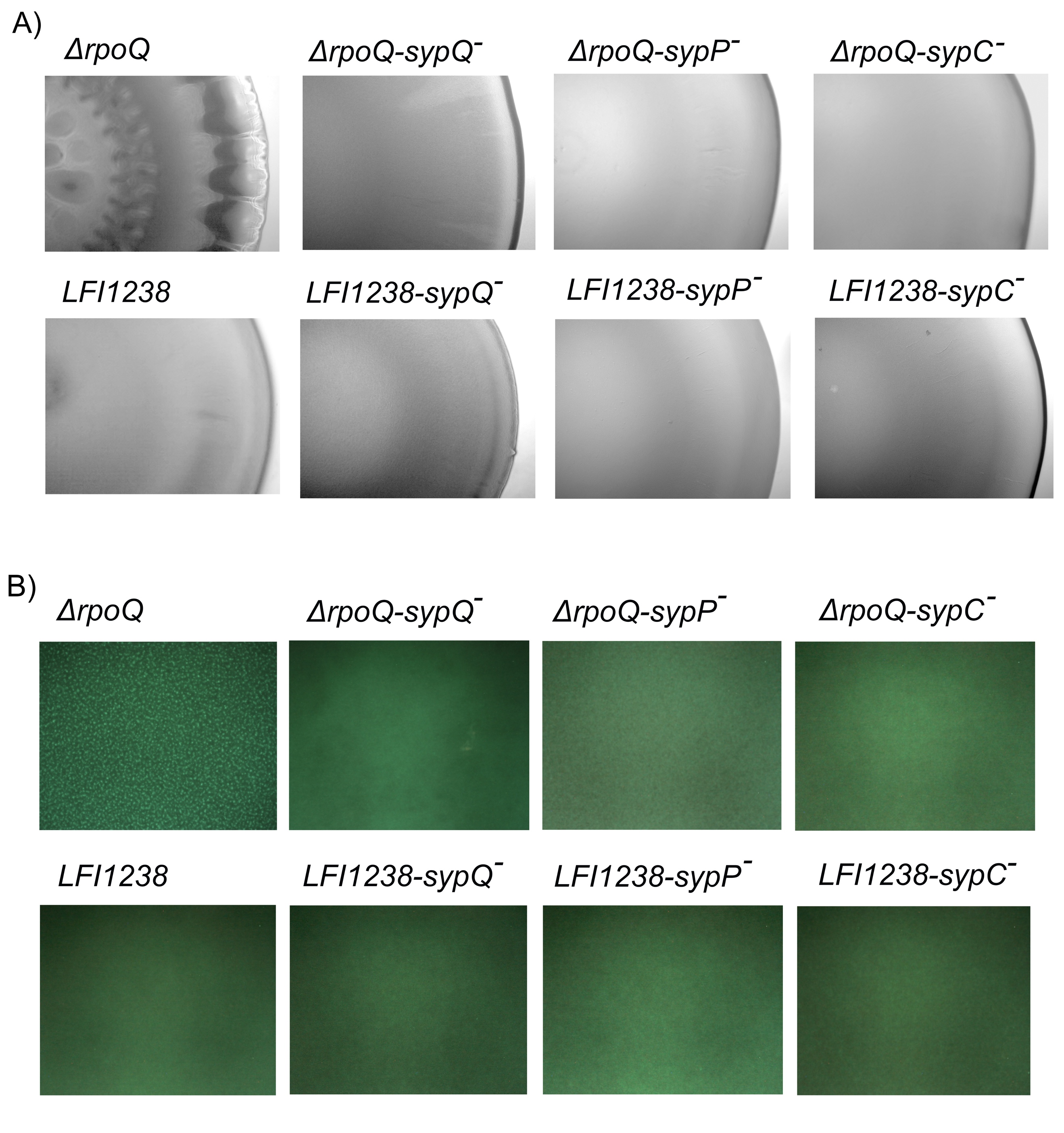


Additional file 10: Figure S1. **Colony morphology and biofilm formation of** *Δ****rpoQ* and LFI1238 *syp* mutants. A)** The colonies of *A. salmonicida* mutant strains (*ΔrpoQ****-****sypQ^-^*, *ΔrpoQ****-****sypP^-^, ΔrpoQ****-****sypC^-^,* LFI1238-*sypQ^-^*, LFI1238-*sypP^-^* and LFI1238-*sypC^-^*) were allowed to form on SWT plates for 12 days at 8°C. The colonies were viewed microscopically with Zeiss Primo Vert and photographed with AxioCam ERc5s at x4 magnification. **B)** The GFP tagged strains (*ΔrpoQ****-****sypQ^-^-*pVSV102, *ΔrpoQ****-****sypP^-^-*pVSV102*, ΔrpoQ****-****sypC^-^-*pVSV102*,* LFI1238-*sypQ^-^-*pVSV102, LFI1238-*sypP^-^-*pVSV102 and LFI1238-*sypC^-^-*pVSV102) were allowed to form biofilms in SWT media at 8°C for 72 hours. The biofilms were viewed, in a Nikon Eclipse TS100 microscope at x10 magnification and photographed with Nikon DS-5Mc.
